# Differential Blood Counts Do Not Consistently Predict Clinical Measurements of Bone Mineral Density and Microarchitecture at Homeostasis

**DOI:** 10.1002/jbm4.10669

**Published:** 2022-08-30

**Authors:** Frederica Schyrr, Pedro Marques‐Vidal, Didier Hans, Olivier Lamy, Olaia Naveiras

**Affiliations:** ^1^ Laboratory of Regenerative Hematopoiesis Swiss Institute for Experimental Cancer Research (ISREC) & Institute of Bioengineering, École Polytechnique Fédérale de Lausanne (EPFL) Lausanne Switzerland; ^2^ Department of Biomedical Sciences University of Lausanne Lausanne Switzerland; ^3^ Lausanne University Hospital and University of Lausanne Lausanne Switzerland; ^4^ Centre of Bone Diseases, Bone and Joint Department Lausanne University Hospital Lausanne Switzerland; ^5^ Hematology Service, Department of Oncology Lausanne University Hospital (CHUV) and University of Lausanne (UNIL) Lausanne Switzerland

**Keywords:** COHORT, CYTOPENIA, HEMATOPOIESIS, OSTEOLAUS, OSTEOPOROSIS

## Abstract

The hematopoietic stem cell niche constitutes a complex bone marrow (BM) microenvironment. Osteoporosis is characterized by both reduced bone mineral density (BMD) and microarchitectural deterioration, constituting the most frequent alteration of the BM microenvironment. It is unclear to which extent modifications of the BM microenvironment, including in the context of osteoporosis, influence blood cell production. We aimed to describe the association between lumbar spine and total hip BMD and microarchitecture (assessed by trabecular bone score [TBS]) and differential blood counts. Data were collected at two time points from 803 (first assessment) and 901 (second assessment) postmenopausal women participating in the CoLaus/OsteoLaus cohort, a population‐based sample in Lausanne, Switzerland. Participants with other active disease or treatment that could influence hematopoiesis or osteoporosis were excluded. Bivariate and multivariate associations between each peripheral blood cell count and BMD or TBS were performed. Additionally, participants in the highest BMD and TBS tertiles were compared with participants in the lowest BMD and TBS tertiles. At first assessment, only neutrophils were significantly different in the lowest BMD and TBS tertile (3.18 ± 0.09 versus 3.47 ± 0.08 G/L, *p* = 0.028). At the second assessment, leucocytes (5.90 ± 0.11 versus 5.56 ± 0.10 G/L, *p* = 0.033), lymphocytes (1.87 ± 0.04 versus 1.72 ± 0.04 G/L *p* = 0.033), and monocytes (0.49 ± 0.01 versus 0.46 ± 0.1 G/L, *p* = 0.033) were significantly different. Power analysis did not identify quasi‐significant associations missed due to sample size. Although significant associations between blood counts and BMD or TBS were found, none was consistent across bone measurements or assessments. This study suggests that, at homeostasis and in postmenopausal women, there is no clinically significant association between the osteoporotic microenvironment and blood production output as measured by differential blood counts. In the context of conflicting reports on the relationship between osteoporosis and hematopoiesis, our study represents the first prospective two time‐point analysis of a large, homogenous cohort at steady state. © 2022 The Authors. *JBMR Plus* published by Wiley Periodicals LLC on behalf of American Society for Bone and Mineral Research.

## Graphical abstract

In the context of conflicting reports on the relationship between osteoporosis and hematopoiesis, this first prospective two timepoint analysis of a large, homogenous cohort at steady state suggests that, at homeostasis and in postmenopausal women, there is no clinically significant association between the osteoporotic microenvironment and blood production output as measured by differential blood counts.

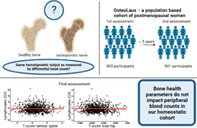

## 1 Introduction

The regulation of hematopoiesis by the local bone marrow (BM) microenvironment has been a subject of extensive research in the last two decades,^(^ ^1^, ^2^, ^3^ ^)^ which has revealed an important link between bone remodeling and hematopoiesis via receptor activator of NF‐κB ligand (RANKL).^(^ ^4^, ^5^ ^)^ Historically, osteolineage cells were the first population described to play a role in hematopoietic stem cell (HSC) regulation through N‐cadherin and parathyroid hormone (PTH)‐mediated expression of Notch ligands.^(^ ^6^, ^7^ ^)^ In fact, osteoblasts were already known determinants of myeloid proliferation through cytokine expression, including granulocyte colony‐stimulating factor (G‐CSF).^(^ ^8^ ^)^ Osteoblasts also produce other soluble mediators such as osteopontin, osteolectin,^(^ ^9^ ^)^ thrombopoietin, and angiopoietin that, while supporting long‐term maintenance of the most primitive HSCs, inhibit overall hematopoiesis through suppression of hematopoietic progenitor proliferation (reviewed in Pino and Frenette^(1^ ^)^). Adipocytes are also abundant components of the adult hematopoietic microenvironment. Although long considered passive space fillers within the bone marrow, they have been shown capable of supporting survival and self‐renewal of the most primitive murine and human HSCs through the secretion of stem cell factor (SCF) and possibly IL‐3,^(^ ^10^, ^11^ ^)^ while inhibiting net hematopoietic progenitor proliferation in the context of either adipocytic conversion of the marrow or committed adipocyte cotransplantation.^(^ ^12^, ^13^ ^)^

A specific perturbation of the BM composition is found in osteoporosis, a disease of the skeleton characterized by reduced bone strength and microarchitectural deterioration. Clinically, dual‐energy X‐ray absorptiometry (DXA) is used to measure areal bone mineral density (BMD g/cm^2^) with results given as a *T*‐score relative to normal values from a healthy about 25‐year‐old population. Osteoporosis is defined by a *T*‐score below −2.5.^(^ ^14^ ^)^ Trabecular bone score (TBS), a gray‐level textural index derived from lumbar spine DXA images, is also used in clinical practice.^(^ ^15^ ^)^ TBS is correlated with parameters reflecting bone microarchitecture and is not influenced by degenerative diseases, including osteoarthritis, as opposed to BMD and thus *T*‐score.^(^ ^16^ ^)^

On a pathophysiological level, an imbalanced activity between osteoblasts and osteoclasts explains the development of osteoporosis.^(^ ^17^ ^)^ Osteoclasts originate from HSCs and are responsible for bone resorption, whereas osteoblasts originate from mesenchymal stromal precursors and secrete the bone matrix.^(^ ^18^ ^)^ The lost equilibrium between osteoblast and osteoclasts in osteoporosis results in an increased bone turnover and an overall loss of trabecular bone. In turn, this bone loss process results in increased adipocyte content in the BM and changes the BM niche.^(^ ^19^, ^20^, ^21^, ^22^, ^23^, ^24^, ^25^ ^)^ As osteoblasts and adipocytes can be derived from the same adult marrow stromal cell (MSC) populations, namely skeletal stem cells, in vitro and in vivo,^(^ ^26^, ^27^ ^)^ it has been proposed that osteoporosis could also be viewed as an imbalanced differentiation of skeletal progenitor cells toward adipocytes,^(^ ^28^ ^)^ a hypothesis that is currently under debate.^(^ ^29^ ^)^ Other studies have proposed a direct contribution of adipocytes to bone loss, both through soluble mediators and direct lipotoxicity on osteoblasts^(^ ^30^ ^)^ (also reviewed in Li and colleagues^(^ ^31^ ^)^).

Thus, osteoporosis reflects a modification in the BM equilibrium of HSC supporting cells, namely osteoblasts, adipocytes, and their common stromal precursors. Accordingly, we set to investigate the impact of modifications in bone microarchitecture, measured by BMD *T*‐score and TBS, on differential blood counts in the postmenopausal population of OsteoLaus, a population‐based sample in Lausanne, Switzerland, after exclusion of a minority of patients predicted to have non‐steady‐state hematopoiesis. Two follow‐up assessments of the cohort were available for analysis. Our overall assumption was that robust, clinically relevant differences should be consistent at both time points and across bone measurements, at the very least resulting in consistent associations between specific blood counts and the two related bone parameters. We specifically hypothesized that a decrease in both BMD *T*‐score and TBS, a measure of bone health, would negatively influence hematopoiesis and associate with lower blood counts (erythrocytes, leucocytes, or platelets).

## 2 Materials and Methods

**Fig. 1:**
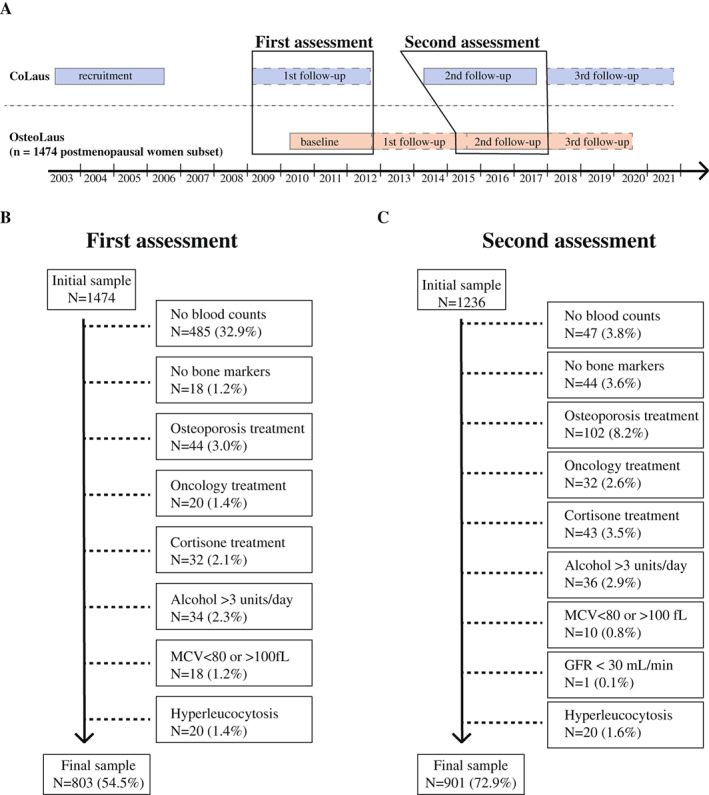
Recruitment process. (*A*) Selection method of the data between CoLaus and OsteoLaus. The first assessment of this study combines the first CoLaus follow‐up with the OsteoLaus baseline data. The second assessment of this study combines the second CoLaus follow‐up with the OsteoLaus second follow‐up data. (*B, C*) Initial sample size and number of participants excluded for final analysis in the first (*B*) and second (*C*) assessments. Percentages represent number of excluded participants over the initial sample. MCV = mean corpuscular volume; GFR = glomerular filtration rate.

OsteoLaus is part of the CoLaus study, a prospective, population‐based study conducted in Lausanne, Switzerland. Details on the recruitment process, characteristics of the population, and assessments of the CoLaus study as well as the OsteoLaus substudy are available elsewhere.^(^ ^32^, ^33^ ^)^ Briefly, the CoLaus study is designed to study cardiovascular risk factors. Recruitment began in June 2003 and ended in May 2006; the first follow‐up was performed between April 2009 and September 2012, the second follow‐up between May 2014 and April 2017, and the third follow‐up between January 2018 and May 2021 (Fig. 1*A*).

The OsteoLaus study includes 1474 postmenopausal women of the CoLaus study.^(^ ^33^ ^)^ Its global aim is to study osteoporosis. OsteoLaus recruitment was conducted between September 2009 and September 2012. Data specific to the OsteoLaus Study were collected during the baseline visit (March 2010 to December 2012) and completed at the first (September 2012 to June 2015), second (March 2015 to February 2018), and third (January 2018 to June 2020) follow‐up visits. A fourth follow‐up visit is ongoing (March 2020 to present). Two assessment periods were selected: the first CoLaus follow‐up was paired with the OsteoLaus baseline data and the second CoLaus follow‐up was paired with the second OsteoLaus follow‐up (Fig. 1*A*).

Data extracted from the CoLaus study included age, body mass index (BMI), alcohol consumption, prescribed and over‐the‐counter drugs, creatinine, C‐reactive protein (CRP), and differential blood counts (hemoglobin, erythrocytes, leucocytes, platelets, neutrophils, lymphocytes, monocytes, basophils, eosinophils). The thresholds used for defining cytopenias for association analysis were as follows: anemia: Hb < 12 g/L; leucopenia: leucocytes <4 G/L; thrombopenia: platelets <150 G/L. Extracted OsteoLaus data included: (i) lumbar spine and total hip BMD expressed as *T*‐score; (ii) TBS adjusted for BMI; (iii) major osteoporotic fracture defined as non‐traumatic vertebral, hip, humerus, or forearm fracture.

High‐sensitivity CRP was assessed by immunoassay (HS latex) with a maximum interassay coefficient of variation of 4.6% and a maximum intra‐assay coefficient of variation of 1.3%. Blood counts were performed on an XE‐2100 apparatus (Sysmex, Horgen, Switzerland) during the first follow‐up of CoLaus and XN‐9000 (Sysmex) for the second follow‐up. DXA scans were performed using Discovery A System (Hologic, Waltham, MA, USA) for all participants at baseline and first follow‐up visits, and Lunar iDXA (GE Healthcare, Madison, WI, USA) at the second follow‐up visit. As the reference population changed, as well as the machine parameters, in order to limit these differences, we worked with *T*‐scores and not with absolute BMD values. Trabecular bone score calculation (TBS iNsight v3.0, Medimaps group, Plan‐les‐Ouates, Geneva, Switzerland) was assessed from the lumbar spine DXA scans. The widely used version of TBS software adjusts the raw TBS value for BMI, as a surrogate for soft tissue thickness via a built‐in algorithm. An experimental version of the software was also used where raw TBS was adjusted for the soft tissue thickness itself.^(^ ^34^ ^)^ BMI‐adjusted TBS was measured prospectively, and adjusted tissue‐thickness TBS retrospectively.

### 2.1 Exclusion criteria

Exclusion criteria were active treatment with chemotherapy, monoclonal antibodies, cortisone, interferon or antiviral drugs (acyclovir, valaciclovir, ganciclovir, tenofovir, lamivudine, entecavir, ribavirin, simeprevir, sofosbuvir, lamivudine, efavirenz), severe renal insufficiency (defined as glomerular filtration rate [GFR] <30 mL/min), alcohol consumption more than 3 drinks/day, abnormal blood parameters suggesting chronic inflammation or underlying constitutional blood disease (hyperleucocytosis: leucocytes ≥10 G/L, mean corpuscular value [MCV] <80 or >100 fl) and any active treatment for osteoporosis except for vitamin D, calcium and hormonal therapy, as well as missing blood counts or missing DXA values.

### 2.2 Statistical analyses

Statistical analysis was performed using Stata version 15.1 for Windows (Stata Corp, College Station, TX, USA). Results are expressed as mean ± standard deviation or Pearson correlation coefficient for bivariate analysis and as multivariable‐adjusted mean ± standard error or standardized beta coefficient for multivariable analysis. Bivariate analysis was performed using chi‐square for categorical variables or Student's *t* test or Kruskal–Wallis test for continuous variables. Multivariate analysis was performed using analysis of variance or linear regression adjusted for age (continuous), BMI (continuous), C‐reactive protein (continuous), and hormonal therapy (yes/no). Results were considered significant if *p* < 0.05.

### 2.3 Ethical statement

The institutional Ethics Committee of the University of Lausanne, which afterward became the Ethics Commission of Canton Vaud (http://www.cer-vd.ch), approved the baseline CoLaus study; the approval was renewed for the subsequent follow‐ups. The full decisions of the CER‐VD can be obtained from the authors upon request. The CoLaus and OsteoLaus studies were performed in agreement with the Helsinki declaration and its former amendments and in accordance with the applicable Swiss legislation. All participants gave their signed informed consent before entering the study.

## 3 Results

### 3.1 Participant selection and characteristics

Of the 1474 postmenopausal participants included in OsteoLaus, final sample size after exclusion criteria including known modifiers of hematopoiesis was 803 at the first assessment and 901 at the second assessment. The exclusion process is outlined in Fig. 1*B, C*. The main reason for exclusion was absence of differential blood counts (32.9% of participants at the first assessment), as this analysis was included well into recruitment and a sizable fraction of the cohort had no data.

At the first assessment, participants were on average 63 years old with a BMI of 25.6 kg/m^2^, 16.9% met osteoporosis definition (minimum BMD *T*‐score of spine or hip ≤ −2.5), and only 15 participants had anemia (1.9%), 29 leukopenia (3.6%), and 5 thrombocytopenia (0.6%). At the second assessment, participants were 67 years old on average, with a BMI of 25.6 kg/m^2^, 13.2% were osteoporotic, and 24 participants presented with anemia (2.7%), 57 leukopenia (6.3%), and 9 thrombocytopenia (1%) (Supplemental Table S1). As expected in an aging cohort, comparison between patients' characteristics at the first and second assessment showed significant differences in age, hemoglobin levels, platelet counts, and leucocyte counts with increased neutrophil and decreased lymphocyte absolute counts (Supplemental Table S2).

### 3.2 Associations between blood count and bone measurements

**Fig. 2:**
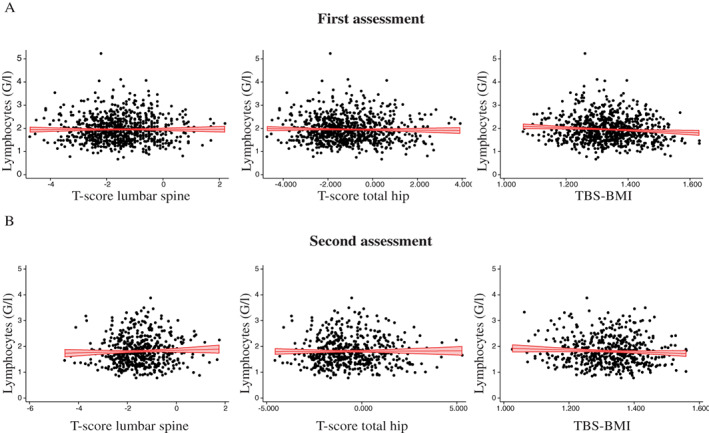
Scatter plot for lymphocyte counts and bone parameters at both assessments. Each dot represents a participant in the first (*A*) and second (*B*) assessments. TBS‐BMI = trabecular bone score adjusted for body mass index. Red indicates regression line with confidence bands.

Several associations were found on a single assessment time point, but only a positive association between neutrophil counts and lumbar spine BMD *T*‐score (Pearson coefficient of 0.1 for the first assessment and 0.1 for the second assessment, bivariate analysis, *p* value <0.05) and a negative association between lymphocyte counts and TBS adjusted for BMI (standardized beta coefficient of −0.12 for the first assessment and −0.16 for the second assessment, multivariate analysis *p* value <0.05) were significant at both assessments. It is important to note that although those associations were consistent between assessments, they were not statistically significant for all three recorded measures of bone health (BMI‐adjusted TBS, *T*‐score lumbar spine, and *T*‐score total hip) as illustrated in Fig. 2 for lymphocyte counts. Remaining statistically significant associations observed were inconsistent between different bone measurements and assessments (Table 1).

**Table 1:** Bivariate and Multivariable Associations Between Bone Measurements and Blood Count, OsteoLaus Study, Lausanne, Switzerland

	TBS‐BMI		*T*‐score lumbar spine		*T*‐score total hip	
	Bivariate	Multivariate	Bivariate	Multivariate	Bivariate	Multivariate
**First assessment**						
Hemoglobin	−0.003	−0.007	0.006	−0.022	0.044	0.008
Erythrocytes	−0.051	−0.021	−0.029	**−0.075**	0.012	−0.031
Leucocytes	−0.057	−0.019	0.062	−0.003	**0.076**	0.008
Platelets	−0.054	−0.049	−0.022	−0.030	−0.025	−0.028
Neutrophils	−0.011	0.048	**0.084**	0.035	**0.088**	0.047
Lymphocytes	**−0.088**	**−0.120**	−0.024	**−0.075**	0.005	−0.070
Monocytes	**−0.098**	−0.055	0.035	−0.001	0.016	−0.014
Basophils	−0.057	−0.044	−0.019	−0.010	−0.026	−0.005
Eosinophils	−0.008	−0.002	0.043	0.024	0.059	0.038
**Second assessment**						
Hemoglobin	−0.050	−0.072	−0.025	−0.026	−0.015	−0.037
Erythrocytes	−0.060	**−0.077**	−0.019	−0.040	0.008	−0.025
Leucocytes	**−0.108**	**−0.139**	**0.077**	0.003	0.046	−0.027
Platelets	−0.010	−0.001	0.030	0.025	−0.002	−0.004
Neutrophils	**−0.100**	**−0.088**	**0.085**	0.025	0.031	−0.015
Lymphocytes	−0.056	**−0.158**	0.004	−0.047	0.037	−0.047
Monocytes	−0.063	−0.056	0.016	−0.041	0.023	−0.016
Basophils	−0.060	−0.056	−0.048	−0.067	**−0.071**	**−0.095**
Eosinophils	−0.029	−0.029	**0.084**	0.060	0.065	0.050

BMI = body mass index; TBS‐BMI = trabecular bone score, adjusted for body mass index.

T‐score total defined as the lowest value between lumbar spine, femoral neck, and hip. Results are expressed as Pearson correlation coefficient (bivariate) and as standardized beta coefficient (multivariable). Multivariate analysis performed by linear regression adjusted for age (continuous), BMI (continuous), C‐reactive protein (continuous), and hormonal therapy (yes/no). Significant (*p* < 0.05) coefficients are indicated in bold.

To further study the impact of BM modification upon osteoporosis, we performed a bivariate and multivariate analysis between categories of bone measurements (pathological versus normal values) and blood counts. The pathological group was defined as participants who presented a BMD *T*‐score below −2.5, which corresponds to the clinical definition of osteoporosis, or a TBS below 1.230, which defines a degraded microarchitecture. At individual assessments, significant associations were present for different combinations of blood cells and bone measurements (basophils with TBS adjusted for BMI, leucocyte and neutrophils with TBS adjusted for total tissue thickness, neutrophils with *T*‐score at the first assessment, as well as erythrocytes, leucocytes, lymphocytes, and monocytes with TBS adjusted for BMI, basophils with lumbar spine *T*‐score or total hip *T*‐score at the second assessment) but none with reproducibility across assessments or consistent across TBS and BMD *T*‐score characteristics (Table 2).

**Table 2:** Bivariate and Multivariate Associations Between Categories of Bone Measurements and Blood Count, OsteoLaus Study, Lausanne, Switzerland

	Bivariate			Multivariate		
	Normal	Degraded	*p* Value	Normal	Degraded	*p* Value
First assessment						
TBS‐BMI (N)	696	107		696	107	
Hemoglobin (g/L)	139 ± 9	139 ± 9	0.405	139 ± 0	139 ± 1	0.454
Erythrocytes (G/L)	4.64 ± 0.32	4.67 ± 0.32	0.338	4.64 ± 0.01	4.65 ± 0.03	0.856
Leucocytes (G/L)	5.94 ± 1.35	5.97 ± 1.25	0.824	5.97 ± 0.05	5.84 ± 0.13	0.366
Platelets (G/L)	257 ± 50	261 ± 56	0.446	257 ± 2	260 ± 5	0.546
Neutrophils (G/L)	3.28 ± 1.02	3.29 ± 0.97	0.914	3.30 ± 0.04	3.16 ± 0.10	0.209
Lymphocytes (G/L)	1.96 ± 0.56	1.94 ± 0.47	0.791	1.96 ± 0.02	1.95 ± 0.05	0.920
Monocytes (G/L)	0.49 ± 0.14	0.50 ± 0.15	0.307	0.49 ± 0.01	0.49 ± 0.01	0.942
Basophils (G/L)	0.03 ± 0.02	0.04 ± 0.02	**0.018**	0.03 ± 0.01	0.04 ± 0.01	**0.034**
Eosinophils (G/L)	0.17 ± 0.12	0.18 ± 0.10	0.338	0.17 ± 0.01	0.18 ± 0.01	0.405
*T*‐score lumbar spine (N)	678	125		678	125	
Hemoglobin (g/L)	139 ± 8	138 ± 10	0.116	139 ± 1	138 ± 1	0.291
Erythrocytes (G/L)	4.64 ± 0.31	4.61 ± 0.36	0.337	4.64 ± 0.01	4.63 ± 0.03	0.670
Leucocytes (G/L)	5.99 ± 1.34	5.70 ± 1.29	**0.026**	5.97 ± 0.05	5.83 ± 0.12	0.285
Platelets (G/L)	257 ± 49	259 ± 61	0.697	257 ± 2	258 ± 5	0.805
Neutrophils (G/L)	3.32 ± 1.01	3.07 ± 1.03	**0.011**	3.31 ± 0.04	3.13 ± 0.09	0.078
Lymphocytes (G/L)	1.95 ± 0.56	1.95 ± 0.52	0.929	1.95 ± 0.02	2.00 ± 0.05	0.348
Monocytes (G/L)	0.49 ± 0.14	0.47 ± 0.13	0.085	0.49 ± 0.01	0.48 ± 0.01	0.307
Basophils (G/L)	0.04 ± 0.02	0.04 ± 0.02	0.734	0.04 ± 0.01	0.04 ± 0.01	0.968
Eosinophils (G/L)	0.17 ± 0.12	0.16 ± 0.11	0.275	0.17 ± 0.01	0.17 ± 0.01	0.551
*T*‐score total hip (N)	667	136		667	136	
Hemoglobin (g/L)	139 ± 8	138 ± 10	0.152	139 ± 0	138 ± 1	0.357
Erythrocytes (G/L)	4.65 ± 0.32	4.61 ± 0.35	0.226	4.64 ± 0.01	4.62 ± 0.03	0.451
Leucocytes (G/L)	5.99 ± 1.34	5.75 ± 1.30	0.056	5.97 ± 0.05	5.86 ± 0.12	0.387
Platelets (G/L)	257 ± 49	260 ± 61	0.504	257 ± 2	259 ± 5	0.601
Neutrophils (G/L)	3.31 ± 1.01	3.13 ± 1.04	0.049	3.30 ± 0.04	3.17 ± 0.09	0.171
Lymphocytes (G/L)	1.96 ± 0.56	1.93 ± 0.51	0.595	1.95 ± 0.02	1.98 ± 0.05	0.541
Monocytes (G/L)	0.49 ± 0.14	0.48 ± 0.13	0.230	0.49 ± 0.01	0.49 ± 0.01	0.538
Basophils (G/L)	0.04 ± 0.02	0.04 ± 0.02	0.659	0.04 ± 0.01	0.04 ± 0.01	0.998
Eosinophils (G/L)	0.17 ± 0.12	0.16 ± 0.11	0.353	0.17 ± 0.01	0.17 ± 0.01	0.643
Second assessment						
TBS‐BMI (N)	745	156		745	156	
Hemoglobin (g/L)	136 ± 9	138 ± 9	0.115	136 ± 0	138 ± 1	**0.060**
Erythrocytes (G/L)	4.55 ± 0.34	4.61 ± 0.36	0.059	4.55 ± 0.01	4.62 ± 0.03	**0.028**
Leucocytes (G/L)	5.78 ± 1.37	6.03 ± 1.48	**0.039**	5.80 ± 0.05	6.04 ± 0.11	0.054
Platelets (G/L)	255 ± 59	255 ± 54	0.976	254 ± 2	254 ± 5	0.980
Neutrophils (G/L)	3.31 ± 1.07	3.44 ± 1.10	0.185	3.34 ± 0.04	3.39 ± 0.09	0.545
Lymphocytes (G/L)	1.78 ± 0.54	1.87 ± 0.60	0.060	1.77 ± 0.02	1.93 ± 0.05	**0.002**
Monocytes (G/L)	0.48 ± 0.14	0.51 ± 0.20	**0.027**	0.48 ± 0.01	0.51 ± 0.01	**0.055**
Basophils (G/L)	0.05 ± 0.03	0.05 ± 0.03	0.058	0.05 ± 0.01	0.05 ± 0.01	0.145
Eosinophils (G/L)	0.16 ± 0.12	0.16 ± 0.10	0.920	0.16 ± 0.01	0.16 ± 0.01	0.747
*T*‐score lumbar spine (N)	815	86		815	86	
Hemoglobin g/L)	137 ± 9	136 ± 10	0.595	137 ± 1	136 ± 1	0.515
Erythrocytes (G/L)	4.57 ± 0.34	4.51 ± 0.4	0.193	4.57 ± 0.01	4.52 ± 0.04	0.191
Leucocytes (G/L)	5.84 ± 1.39	5.69 ± 1.44	0.344	5.83 ± 0.05	5.91 ± 0.16	0.657
Platelets (G/L)	255 ± 57	254 ± 65	0.950	254 ± 2	252 ± 7	0.688
Neutrophils (G/L)	3.35 ± 1.07	3.23 ± 1.11	0.336	3.35 ± 0.04	3.34 ± 0.12	0.924
Lymphocytes (G/L)	1.80 ± 0.55	1.76 ± 0.56	0.571	1.79 ± 0.02	1.86 ± 0.06	0.291
Monocytes (G/L)	0.48 ± 0.15	0.48 ± 0.14	0.951	0.48 ± 0.01	0.50 ± 0.02	0.427
Basophils (G/L)	0.05 ± 0.03	0.05 ± 0.03	**0.001**	0.05 ± 0.01	0.05 ± 0.01	**0.002**
Eosinophils (G/L)	0.16 ± 0.12	0.15 ± 0.10	0.438	0.16 ± 0.01	0.16 ± 0.01	0.540
*T*‐score total hip (N)	782	119		782	119	
Hemoglobin (g/L)	137 ± 9	136 ± 9	0.231	137 ± 0	136 ± 1	0.398
Erythrocytes (G/L)	4.57 ± 0.34	4.51 ± 0.37	0.070	4.57 ± 0.01	4.52 ± 0.03	0.194
Leucocytes (G/L)	5.85 ± 1.39	5.64 ± 1.41	0.117	5.84 ± 0.05	5.85 ± 0.14	0.928
Platelets (G/L)	255 ± 58	255 ± 60	0.909	254 ± 2	253 ± 6	0.875
Neutrophils (G/L)	3.36 ± 1.07	3.21 ± 1.10	0.182	3.35 ± 0.04	3.33 ± 0.10	0.898
Lymphocytes (G/L)	1.80 ± 0.55	1.75 ± 0.56	0.301	1.80 ± 0.02	1.83 ± 0.05	0.604
Monocytes (G/L)	0.48 ± 0.16	0.47 ± 0.13	0.457	0.48 ± 0.01	0.49 ± 0.02	0.891
Basophils (G/L)	0.05 ± 0.03	0.05 ± 0.03	**0.031**	0.05 ± 0.01	0.05 ± 0.01	**0.033**
Eosinophils (G/L)	0.16 ± 0.12	0.15 ± 0.09	0.155	0.16 ± 0.01	0.15 ± 0.01	0.285

BMI = body mass index; TBS‐BMI = trabecular bone score, adjusted for body mass index.

For TBS, degraded microarchitecture defined for a TBS below 1.230; osteoporosis defined for a *T*‐score below −2.5. Results are expressed as mean ± standard deviation for bivariate analysis and as multivariate‐adjusted mean ± standard error for multivariate analysis. Between‐group comparisons performed using analysis of variance. Multivariate analysis adjusted for age (continuous), BMI (continuous), C‐reactive protein (continuous), and hormonal therapy (yes/no).

### 3.3 Comparison between tertiles of bone measurements

Next, to investigate the effect of osteoporosis on blood counts in the participants with the most extreme bone remodeling, we performed a comparison between participants included in the highest tertile of BMD *T*‐score as well as TBS and participants in the lowest tertile for BMD *T*‐score and TBS. Neutrophils were significantly different in the lowest than the highest tertile at the first assessment. At the second assessment, significant differences were found for leucocytes, lymphocytes, and monocytes, but not for neutrophil counts (Table 3).

**Table 3:** Comparison Between Participants in the Lowest Tertiles and Participants in the Highest Tertiles of Bone Mineral Density and Trabecular Bone Score, OsteoLaus Study, Lausanne, Switzerland

	Lowest	Highest	*p* Value
First assessment, sample size	160	173	
Hemoglobin (g/L)	138 ± 1	140 ± 1	0.169
Erythrocytes (G/L)	4.63 ± 0.03	4.64 ± 0.03	0.683
Leucocytes (G/L)	5.86 ± 0.11	6.11 ± 0.11	0.130
Platelets (G/L)	260 ± 5	260 ± 4	0.932
Neutrophils (G/L)	3.18 ± 0.09	3.47 ± 0.08	**0.028**
Lymphocytes (G/L)	1.98 ± 0.05	1.92 ± 0.04	0.337
Monocytes (G/L)	0.49 ± 0.01	0.50 ± 0.01	0.391
Basophils (G/L)	0.03 ± 0.01	0.04 ± 0.01	0.613
Eosinophils (G/L)	0.16 ± 0.01	0.17 ± 0.01	0.197
Second assessment, sample size	174	185	
Hemoglobin (g/L)	137 ± 1	136 ± 1	0.281
Erythrocytes (G/L)	4.59 ± 0.03	4.53 ± 0.03	0.123
Leucocytes (G/L)	5.90 ± 0.11	5.56 ± 0.10	**0.033**
Platelets (G/L)	252 ± 4	258 ± 4	0.363
Neutrophils (G/L)	3.33 ± 0.08	3.15 ± 0.08	0.173
Lymphocytes (G/L)	1.87 ± 0.04	1.72 ± 0.04	**0.033**
Monocytes (G/L)	0.49 ± 0.01	0.46 ± 0.01	**0.033**
Basophils (G/L)	0.05 ± 0.01	0.04 ± 0.01	0.109
Eosinophils (G/L)	0.16 ± 0.01	0.16 ± 0.01	0.917

Only participants who were in the lowest (respectively highest) tertiles of bone mineral density and trabecular bone score were included. Results are expressed as multivariate‐adjusted mean ± standard error. Between‐group comparisons performed using analysis of variance adjusted for age (continuous), BMI (continuous), C‐reactive protein (continuous), and hormonal therapy (yes/no).

To complete the investigation on the effect of osteoporosis in blood counts for participants with the most extreme bone remodeling, we performed a cross‐sectional analysis of major osteoporotic fracture and blood counts. Both at the first and second assessments, the eosinophil count was higher among the participants who had or developed a major fracture (Supplemental Table S3). Other parameters did not show consistent association with major osteoporotic fractures.

Finally, power analysis was performed to investigate the possibility of missing a clinically significant association (Supplemental Table S4). Sample sizes that would have been required to uncover associations between bone measurements and blood counts missed due to underpower were mostly above 5000. Therefore, power analysis did not identify quasi‐significant associations missed due to sample size.

## 4 Discussion

In the OsteoLaus cohort, we found no consistent association between bone health and circulating blood cell counts. Our study supports the recent results of the prospective observational cohort in the Cardiovascular Health Study, which found no association by linear regression analysis between hemoglobin levels and BMD (total hip, lumbar spine, or total body) nor by Poisson regression between anemia and low BMD in the analytic cohort of 1513 men and women aged >65 years.^(^ ^35^ ^)^ We extend those results to all circulating blood cell lineages, namely platelets and leucocytes with differential counts, and include a second set of bone parameters, the TBS, in the context of a postmenopausal female cohort of similar size.

Our cohort was analyzed at two time points separated by 3 to 6 years. The evolution of blood counts and bone parameters across the two assessments reflected the expected differences of an aging cohort. Specifically, lumbar spine BMD *T*‐score increased, as a reflection of the degenerative process happening in aging vertebras.^(^ ^16^ ^)^ Contrarily, both total hip BMD *T*‐score and TBS showed a significant decrease in bone health, the latter due to its robustness to degenerative changes.^(^ ^16^ ^)^ Regarding the blood counts, hemoglobin slightly but significantly decreased, as expected.^(^ ^36^ ^)^ Within the white blood cells, neutrophils significantly increased, and lymphocytes significantly decreased, reflecting the known myeloid bias imposed by aging.^(^ ^37^ ^)^ Thus, our cohort of postmenopausal women displays expected characteristics of an aging cohort.

However, our analysis of association between blood counts and bone measurements failed to find any consistent, statistically significant results. We used bone measurements as continuous as well as categorical variables, first to reflect a phenotype and then to address a clinically relevant question. Both types of analysis were negative. Of note, the experimental TBS adjusted for total tissue thickness^(^ ^34^ ^)^ was examined for all our analysis and showed similar results as TBS adjusted for BMI (data not shown). To uncover an effect that would be limited to extreme modifications of the bone architecture, we performed an analysis between the participants with healthiest bone measurements compared with the participants with the most degraded bone measurements, which failed again to show any consistent and significant effect on peripheral blood counts. Furthermore, no other consistent, statistically significant association was found between blood cell counts and prevalent major osteoporotic fractures.

Several studies, as summarized in Supplemental Table S5, have investigated the relationship between osteoporosis and circulating blood cells in otherwise healthy participants. In the early 2000s, one group reported a positive association between total lymphocyte counts and BMD in two different cohorts of women—a postmenopausal one composed of 124 participants and a hip‐fractured cohort of 176 participants.^(^ ^38^, ^39^ ^)^ In 2010, no difference in blood cell counts was found between 26 women with osteoporotic fracture and 24 healthy controls.^(^ ^40^ ^)^ Subsequently, different groups focused on a possible link between anemia and osteoporosis and found a positive association in both 371 postmenopausal Turkish women^(^ ^41^ ^)^ and in two Italian older population‐based cohorts composed of 358 and 950 participants, respectively.^(^ ^42^, ^43^ ^)^ While examining the link between osteoporosis and the neutrophil lymphocyte ratio, a retrospective study in Turkey also found a positive correlation between hemoglobin and BMD.^(^ ^44^ ^)^ More recently, in 2589 older American men, an association between anemia, low lymphocyte counts, high neutrophil counts, and bone density loss was described.^(^ ^45^ ^)^ In 2325 Chinese men older than 50 years, participants with at least osteopenia had lower red blood cell, mononuclear cell, and neutrophil counts than controls but higher platelet counts.^(^ ^46^ ^)^ In a cohort of 33 Korean healthy postmenopausal women, a positive association between white blood cells, red blood cells, as well as platelets and BMD *T*‐score was also found.^(^ ^47^ ^)^ Also in Korea, using two different cohorts, a negative correlation was found between platelets and BMD.^(^ ^48^ ^)^ A negative association between platelets and BMD was also found in a Swedish prospective along with a positive correlation with hemoglobin, a negative one with neutrophils, and no significant correlation with white blood cells.^(^ ^49^ ^)^ Previously, we also described a positive association between neutrophils and platelet and BMD *T*‐score in a cohort of 143 breast cancer patients undergoing chemotherapy.^(^ ^50^ ^)^ Thus, a clear and congruent effect of osteoporosis on specific blood cell counts could not be highlighted across previous studies even if high loss of BMD showed more convincing associations.

Our current study shows no consistent association between BMD measurements and steady‐state blood cell counts. As outlined above, based on previous literature, our findings in our postmenopausal women cohort at steady state are discordant. This may reflect the fact that the population in OsteoLaus, at age 63.1 ± 7.8 and 67.8 ± 7.7, respectively, for the two assessments, is relatively younger and healthier than other studies reporting specific associations between bone health parameters and cell blood counts (cohort comparisons detailed in Supplemental Table S5). Therefore, deficiencies in the BM microenvironment may be less pronounced in our cohort. Specifically, 16.9% of participants in the OsteoLaus cohort met the definition of osteoporosis, as compared with other reported cohorts where average prevalence of osteoporosis was closer to 30% to 50%.^(^ ^40^, ^42^, ^44^, ^46^, ^48^, ^50^ ^)^ Similarly, only 1.9% and 2.7% of participants met, respectively, the definition of anemia (Hb < 12 g/L) in the first and second assessment in OsteoLaus, as opposed to 22% in the similarly aged Turkish postmenopausal cohort.^(41^ ^)^ The OsteoLaus cohort also presented lower prevalence of diabetes, 4.1%,^(^ ^33^ ^)^ versus 17.25% in the Turkish cohort, which additionally reported average BMI > 30 kg/m^2^.^(41^ ^)^ Furthermore, although the Korean cohort reported by Kim and colleagues^(^ ^47^ ^)^ constitutes our most comparable postmenopausal cohort in terms of age, BMI, and red blood cell counts, women on hormone replacement therapy (HRT) or vitamin D were excluded for the Korean study. Contrarily, OsteoLaus participants could take vitamin D substitutes, were shown to have adequate plasma 25‐hydroxyvitamin D levels, and 54% were on HRT at the first assessment, an intervention shown to be beneficial to BMD even after withdrawal.^(^ ^51^ ^)^ Although HRT in postmenopausal women has not been generally associated with changes in circulating cell blood counts, some studies report higher B lymphocyte counts.^(^ ^52^, ^53^ ^)^ It is also important to note that our cohort is composed of only women, and it would be possible that the impact of bone health on blood counts may be more prominent in men.

A further hypothesis to explain the observed discordance between our study and the literature is that every publication described an association with at least one parameter. However, there is no consistency between studies. This could potentially be explained by reporting bias, with only publishing significant associations and giving the impression of a convincing effect of osteoporosis on blood counts. Our data also showed some significant associations at each assessment but those associations were not stable over time. All other publications only described one time point and therefore could not confirm the effect found over time. This might have led to an overinterpretation of the observed associations, whereas our study and the one by Valdemorro and colleagues^(^ ^35^ ^)^ were more stringent and might explain our negative results. Furthermore, animal models of osteoporosis also failed to uncover any modification in blood counts at steady state.^(^ ^54^, ^55^ ^)^ Biologically, the lack of association between bone health and blood counts is important as it suggests that BM has the capacity to compensate for modifications in its composition and microarchitecture without compromising blood cell output. Clinically, our study indicates that blood counts alone cannot be used as a surrogate marker to assess bone health and cannot guide the decision to perform DXA measurement in otherwise healthy postmenopausal women.

Our study presents different strengths. Because of its large size and homogeneity and as outlined by the power analysis performed, clinically relevant associations were unlikely to be missed. For example, the sample size needed to consider the beta coefficient between a bone measurement and hemoglobin as significant, at the first time point, was more than 15,000 participants. In this context, a clinically and biologically relevant effect of osteoporosis on hemoglobin in homeostasis is improbable. However, the trend for the association between major osteoporotic fractures and anemia at both time points (Supplemental Table S3 *A*, *B*), in the absence of statistical association between blood counts and bone health parameters, suggests that the clinical constellation of pathological red blood cell levels and bone fractures may be more relevant than the relationship between the measured biomarkers. Additionally, it is important to note as compared with other published cohorts that CoLaus and OsteoLaus are large studies performed in a predefined way with professionals dedicated to data collection. It ensures a robust and reliable data set and multiple time point analysis. In addition, two types of bone parameters are included in OsteoLaus; the standard BMD *T*‐score measured by DXA and also TBS. This new marker has the advantage of not being influenced by age‐related degenerative disease in contrast to BMD.^(^ ^16^ ^)^

We also encountered several limitations. First, blood counts and DXA scan were not performed on the same day because of the inherent nature of the study. Although this is an important element to consider, both analyses were performed within an average of 6 months. Biologically, osteoporosis is a slow process, with a decrease of 1.9% in lumbar spine BMD per year described in postmenopausal women.^(^ ^56^ ^)^ Furthermore, women in the CoLaus cohort were asked to postpone their visit if they presented any sign of infection, had had recent surgery, or were generally feeling unwell. In those conditions, blood counts can be interpreted to represent steady state. Additionally, patients with elevated CRP, suggesting an active infection or inflammatory process, were excluded. Thus, even though blood sampling and DXA measurement were not conducted at the exact same time, the slow process of bone modification and a blood count representing steady state allow a combination of those values to represent one time point. In addition, BMD measurements were performed on two different machines, which may have interfered with obtaining similar results at both time points since *T*‐scores were used to classify bone health categories. However, it allows for our finding to be generally applicable and not limited by technique.

Second, in keeping with the fact that participants only came to visit if free of any acute event, our cohort is limited to a healthy, homeostatic group. Therefore, our findings cannot be extrapolated to disordered bone health or hematopoiesis, even if patients with a hematopoietic disorder not affecting MCV and not leading to leukocytosis were not actively excluded. Furthermore, CoLaus focuses on a Swiss predominantly Caucasian population and the OsteoLaus cohort is designed to study osteoporosis in postmenopausal women and *per se* excludes male participants and is limited to the 55‐ to 75‐year‐old age range of the participants in the cohort. Therefore, our study has limited generalization potential to a more diverse population. Its homogeneity also prevents the identification of particular stress situations that might uncover an effect of osteoporosis in hematopoiesis. Moreover, BM biopsies or aspirate were not performed on OsteoLaus participants and our interpretation thus infers hematopoietic function by output without the capacity of analyzing the compensatory effects within the BM.

Finally, our study aimed at uncovering robust associations between blood counts and bone parameters. Finer associations limited to bone microarchitecture (eg, TBS) and a simple blood lineage (eg, lymphocyte decline) have been interpreted as non‐consistent in our study but may reflect the pathophysiology of the osteogenic niche, as described in mice for B‐cell lymphopoiesis.^(^ ^9^, ^57^ ^)^

While extensive and conducted in a large cohort, our study prompts further research in situations where hematopoiesis is under stress, as it is the case, for example, during chemotherapy treatment or in presence of moderate to profound cytopenias. Faster proliferation of hematopoietic cells could uncover their need for a specific supportive BM microenvironment and possibly to investigate such needs in osteoporosis. Our study underlines the complexity of understanding the clinical relevance of changes in the BM cellularity and bone microarchitecture.

In this cohort of postmenopausal women assessed at two separate time points and with two different DXA instruments, bone health did not have a reproductible impact on peripheral blood cell counts at homeostasis. This study underlines the complexity of BM regulation, such that osteoporotic modifications of the bone marrow microenvironment do not impact peripheral blood counts, and thus hematopoietic stem and progenitor cell final output, in steady‐state conditions.

## Disclosures

All authors state that they have no conflicts of interest.

## Author Contributions

**Frederica Schyrr:** Conceptualization; funding acquisition; investigation; methodology; visualization; writing – original draft. **Pedro Marques‐Vidal:** Data curation; formal analysis; funding acquisition; investigation; methodology; resources; software; visualization; writing – review and editing. **Didier Hans:** Formal analysis; methodology; resources; software; validation; writing – review and editing. **Olivier Lamy:** Conceptualization; funding acquisition; investigation; methodology; project administration; resources; supervision; validation; writing – review and editing. **Olaia Naveiras:** Conceptualization; funding acquisition; investigation; project administration; supervision; validation; visualization; writing – review and editing.

### Peer Review

The peer review history for this article is available at https://publons.com/publon/10.1002/jbm4.10669.

## Supporting information


**Table S1**. Comparison between included and excluded participants of the OsteoLaus study, Lausanne, Switzerland
**Table S2.** Comparison between participants' characteristics at the first and second assessment, OsteoLaus study, Lausanne, Switzerland
**Table S3.** (*A*) Comparison between participants with or without low‐trauma major osteoporotic fracture at the first assessment
**Table S3.** (*B*) Comparison between participants with or without low‐trauma major osteoporotic fracture at the second assessment
**Table S4.** Sample size needed to consider the beta coefficient between a bone and blood marker as significant, OsteoLaus study, Lausanne, Switzerland
**Table S5.** Review of the available literature regarding correlation between bone health parameters and blood cell counts in osteoporotic participants

## Data Availability

The data that support the findings of this study are available upon request to the CoLaus consortium https://www.colaus-psycolaus.ch/professionals/how-to-collaborate/
