# Supplementary material for: Differential Blood Counts Do Not Consistently Predict Clinical Measurements of Bone Mineral Density and Microarchitecture at Homeostasis
Source: JBMR Plus. 2022 Aug 30;6(9):e10669. doi: 10.1002/jbm4.10669 (PMC9464992; doi:10.1002/jbm4.10669)
Supplement: Supplementary file 1 — Table S1. Comparison between included and excluded participants of the OsteoLaus study, Lausanne, Switzerland Table S2. Comparison between participants' characteristics at the first and second assessment, OsteoLaus study, Lausanne, Switzerland Table S3. (A) Comparison between participants with or without low‐trauma major osteoporotic fracture at the first assessment Table S3. (B) Comparison between participants with or without low‐trauma major osteoporotic fracture at the second assessment Table S4. Sample size needed to consider the beta coefficient between a bone and blood marker as significant, OsteoLaus study, Lausanne, Switzerland Table S5. Review of the available literature regarding correlation between bone health parameters and blood cell counts in osteoporotic participants [file JBM4-6-e10669-s001.docx]

**Appendix :**

**Supplementary data:**

- **Supplementary table 1 :** Comparison between included and excluded participants of the OsteoLaus study, Lausanne, Switzerland.
- **Supplementary table 2 :** Comparison between participants’ characteristics at the first and second assessment, OsteoLaus study, Lausanne, Switzerland.
- **Supplementary table 3A :** Comparison between participants with or without low trauma major osteoporotic fracture at the first assessment
- **Supplementary table 3B :** Comparison between participants with or without low trauma major osteoporotic fracture at the second assessment
- **Supplementary table 4 :** Sample size needed to consider the beta coefficient between a bone and blood marker as significant, OsteoLaus study, Lausanne, Switzerland.
- **Supplementary table 5 :** Review of the available literature regarding correlation between bone health parameters and blood cell counts in osteoporotic participants.

**Supplementary table 1.** Comparison between included and excluded participants, the OsteoLaus study, Lausanne, Switzerland.

|  | **First assessment** | |  | **Second assessment** | |  |
| --- | --- | --- | --- | --- | --- | --- |
|  | **Included** | **Excluded** | **P-value** | **Included** | **Excluded** | **P-value** |
| Sample size | 803 | 664 |  | 901 | 462 |  |
| Age (years) | 63.1 ± 7.8 | 63.9 ± 8.1 | 0.051 | 67.8 ± 7.7 | 70.4 ± 8.2 | <0.001 |
| BMI (kg/m^2^) | 25.65 ± 4.5 | 25.57 ± 4.49 | 0.726 | 25.69 ± 4.60 | 26.41 ± 4.83 | 0.0084 |
| Bone measurements |  |  |  |  |  |  |
| BMD T-score lumbar spine | -1.11 ± 1.43 | -1.07 ± 1.51 | 0.621 | -0.59 ± 1.67 | -0.75 ± 1.55 | 0.136 |
| TBS-BMI | 1.33 ± 0.10 | 1.32 ± 0.10 | 0.008 | 1.32 ± 0.09 | 1.30 ± 0.10 | 0.028 |
| Blood markers |  |  |  |  |  |  |
| Haemoglobin (g/l) | 139 ± 9 | 137 ± 10 | 0.041 | 136 ± 9 | 136 ± 11 | 0.397 |
| Erythrocytes (G/l) | 4.64 ± 0.32 | 4.62 ± 0.47 | 0.507 | 4.56 ± 0.34 | 4.55 ± 0.45 | 0.666 |
| Leucocytes (G/l) | 5.95 ± 1.34 | 6.95 ± 4.30 | <0.001 | 5.82 ± 1.39 | 6.75 ± 3.98 | <0.001 |
| Platelets (G/l) | 257 ± 51 | 257 ± 60 | 0.975 | 255 ± 58 | 256 ± 65 | 0.670 |
| Neutrophils (G/l) | 3.28 ± 1.02 | 3.82 ± 2.27 | <0.001 | 3.34 ± 1.07 | 3.92 ± 1.84 | 0.001 |
| Lymphocytes (G/l) | 1.95 ± 0.55 | 2.05 ± 0.89 | 0.052 | 1.80 ± 0.55 | 1.98 ± 2.67 | 0.053 |
| Monocytes (G/l) | 0.49 ± 0.14 | 0.53 ± 0.19 | 0.005 | 0.48 ± 0.15 | 0.54 ± 0.23 | <0.001 |
| Basophils (G/l) | 0.04 ± 0.02 | 0.04 ± 0.02 | 0.414 | 0.05 ± 0.03 | 0.05 ± 0.05 | 0.426 |
| Eosinophils (G/l) | 0.17 ± 0.12 | 0.16 ± 0.10 | 0.333 | 0.16 ± 0.12 | 0.17 ± 0.13 | 0.153 |
| Anaemia (%) | 15 (1.9) | 6 (3.2) | 0.247 | 24 (2.7) | 23 (5.5) | 0.009 |
| Leukopenia (%) | 29 (3.6) | 5 (2.7) | 0.533 | 57 (6.3) | 20 (4.8) | 0.271 |
| Thrombocytopenia (%) | 5 (0.6) | 3 (1.6) | 0.177 ǂ | 9 (1) | 9 (2.2) | 0.092 |
| Hyperleucocytosis (%) | 0 (0) | 24 (12.9) | <0.001 | 0 (0) | 38 (9.1) | <0.001 |
| CRP (mg/L) | 2.35 ± 2.89 | 2.72 ± 3.47 | 0.086 § | 2.00 ± 2.31 | 2.30 ± 2.76 | 0.172 § |

BMI, body mass index; CRP, C-reactive protein; TBS-BMI, trabecular bone score, adjusted for body mass index; Results are expressed as number of participants (%) for categorical variables or as mean ± standard deviation for continuous variables. Between group comparisons performed using chi-square for categorical variables or student t-test or Kruskal-Wallis test (§) for continuous variables.

**Supplementary table 2.** Comparison between participants’ characteristics at the first and second assessment, OsteoLaus study, Lausanne, Switzerland.

|  | **First assessment** | **Second assessment** | **P-value** |
| --- | --- | --- | --- |
| Sample size | 549 | 549 |  |
| BMI (kg/m^2^) | 25.4 ± 4.5 | 25.9 ± 4.6 | <0.001 |
| Bone measurements |  |  |  |
| BMD T-score lumbar spine | -1.11 ± 1.36 | -0.67 ± 1.57 | <0.001 |
| TBS, BMI | 1.34 ± 0.1 | 1.32 ± 0.09 | <0.001 |
| Blood markers |  |  |  |
| Haemoglobin (g/l) | 139 ± 8 | 137 ± 9 | <0.001 |
| Erythrocytes (G/l) | 4.65 ± 0.30 | 4.57 ± 0.34 | <0.001 |
| Leucocytes (G/l) | 5.82 ± 1.3 | 5.92 ± 1.4 | 0.042 |
| Platelets (G/l) | 257 ± 50 | 253 ± 56 | 0.006 |
| Neutrophils (G/l) | 3.2 ± 1.01 | 3.39 ± 1.09 | <0.001 |
| Lymphocytes (G/l) | 1.93 ± 0.54 | 1.82 ± 0.55 | <0.001 |
| Monocytes (G/l) | 0.48 ± 0.14 | 0.5 ± 0.16 | <0.001 |
| Basophils (G/l) | 0.04 ± 0.02 | 0.05 ± 0.03 | <0.001 |
| Eosinophils (G/l) | 0.17 ± 0.11 | 0.17 ± 0.12 | 0.854 |
| CRP | 2.29 ± 2.69 | 1.93 ± 2.34 | <0.001(§) |

BMI, body mass index; CRP, C-reactive protein; TBS-BMI, trabecular bone score, adjusted for body mass index; TBS-TTH, trabecular bone score, adjusted for total tissue thickness. Participants with available values at both assessments were included for this analysis. Results are expressed as mean ± standard deviation. Between group comparisons performed using student t-test or Kruskal-Wallis test (§) for CRP.

**Supplementary Table 3A** Comparison between participants with or without low trauma major osteoporotic fracture at the first assessment

|  | **Bivariate** | |  | **Multivariate** | |  |
| --- | --- | --- | --- | --- | --- | --- |
|  | **No fracture** | **Fracture** | **P-value** | **No fracture** | **Fracture** | **P-value** |
| Sample size | 735 | 68 |  | 735 | 68 |  |
| Blood markers |  |  |  |  |  |  |
| Haemoglobin (g/l) | 139 ± 9 | 138 ± 10 | 0.227 | 139 ± 1 | 138 ± 1 | 0.291 |
| Erythrocytes (G/l) | 4.64 ± 0.32 | 4.64 ± 0.37 | 0.967 | 4.64 ± 0.01 | 4.64 ± 0.04 | 0.919 |
| Leucocytes (G/l) | 5.96 ± 1.35 | 5.85 ± 1.25 | 0.510 | 5.96 ± 0.05 | 5.86 ± 0.16 | 0.540 |
| Platelets (G/l) | 257 ± 51 | 261 ± 49 | 0.516 | 257 ± 2 | 261 ± 6 | 0.485 |
| Neutrophils (G/l) | 3.29 ± 1.03 | 3.17 ± 0.92 | 0.350 | 3.29 ± 0.04 | 3.14 ± 0.12 | 0.224 |
| Lymphocytes (G/l) | 1.96 ± 0.55 | 1.92 ± 0.55 | 0.647 | 1.96 ± 0.02 | 1.97 ± 0.07 | 0.828 |
| Monocytes (G/l) | 0.49 ± 0.14 | 0.50 ± 0.15 | 0.711 | 0.49 ± 0.01 | 0.49 ± 0.02 | 0.984 |
| Basophils (G/l) | 0.03 ± 0.02 | 0.04 ± 0.02 | 0.050 | 0.03 ± 0.01 | 0.04 ± 0.01 | 0.113 |
| Eosinophils (G/l) | 0.17 ± 0.12 | 0.20 ± 0.11 | 0.056 | 0.17 ± 0.01 | 0.20 ± 0.01 | 0.046 |
| Anaemia (%) | 11 (1.5) | 4 (5.9) | 0.031 | 1 (ref.) | 3.35 (0.99 - 11.3) | 0.052 |
| Leukopenia (%) | 27 (3.7) | 2 (2.9) | 1.000 | 1 (ref.) | 0.84 (0.19 - 3.71) | 0.820 |
| Thrombocytopenia (%) | 5 (0.7) | 0 (0) | 1.000 | 1 (ref.) | NC |  |
| Hyperleucocytosis (%) | 0 (0) | 0 (0) | NC | 1 (ref.) | NC |  |

NC, not computable. For bivariate analyses, results are expressed as number of participants (%) for categorical variables or as mean ± standard deviation for continuous variables. Between group comparisons performed using Fisher’s exact test for categorical variables or student t-test for continuous variables. For multivariate analysis, results are expressed as adjusted odd ratio and (95% confidence interval) for categorical variables and as mean ± standard error for continuous variables. Comparisons performed using logistic regression for categorical variables and analysis of variance for continuous variables. Multivariate models adjusted on age, body mass index and CRP.

**Supplementary Table 3B** Comparison between participants with or without low trauma major osteoporotic fracture at second assessment

|  | **Bivariate** | |  | **Multivariate** | |  |
| --- | --- | --- | --- | --- | --- | --- |
|  | **No fracture** | **Fracture** | **P-value** | **No fracture** | **Fracture** | **P-value** |
| Sample size | 833 | 68 |  | 833 | 68 |  |
| Blood markers |  |  |  |  |  |  |
| Haemoglobin (g/l) | 136 ± 9 | 137 ± 10 | 0.602 | 137 ± 0 | 137 ± 1 | 0.552 |
| Erythrocytes (G/l) | 4.56 ± 0.34 | 4.53 ± 0.40 | 0.425 | 4.57 ± 0.01 | 4.52 ± 0.04 | 0.316 |
| Leucocytes (G/l) | 5.79 ± 1.38 | 6.24 ± 1.46 | 0.010 | 5.81 ± 0.05 | 6.22 ± 0.17 | 0.023 |
| Platelets (G/l) | 255 ± 59 | 249 ± 51 | 0.411 | 255 ± 2 | 247 ± 7 | 0.292 |
| Neutrophils (G/l) | 3.32 ± 1.07 | 3.56 ± 1.10 | 0.073 | 3.33 ± 0.04 | 3.51 ± 0.13 | 0.199 |
| Lymphocytes (G/l) | 1.79 ± 0.54 | 1.91 ± 0.66 | 0.098 | 1.79 ± 0.02 | 1.94 ± 0.07 | 0.036 |
| Monocytes (G/l) | 0.48 ± 0.15 | 0.52 ± 0.13 | 0.047 | 0.48 ± 0.01 | 0.51 ± 0.02 | 0.143 |
| Basophils (G/l) | 0.05 ± 0.03 | 0.06 ± 0.02 | 0.003 | 0.05 ± 0.01 | 0.06 ± 0.01 | 0.004 |
| Eosinophils (G/l) | 0.16 ± 0.12 | 0.19 ± 0.13 | 0.034 | 0.16 ± 0.01 | 0.19 ± 0.01 | 0.072 |
| Anaemia (%) | 20 (2.4) | 4 (5.9) | 0.100 | 1 (ref.) | 1.99 (0.62 - 6.34) | 0.245 |
| Leukopenia (%) | 54 (6.5) | 3 (4.4) | 0.794 | 1 (ref.) | 0.58 (0.13 - 2.47) | 0.458 |
| Thrombocytopenia (%) | 9 (1.1) | 0 (0) | 1.000 | 1 (ref.) | NC |  |
| Hyperleucocytosis (%) | 0 (0) | 0 (0) | NC | 1 (ref.) | NC |  |

NC, not computable. For bivariate analyses, results are expressed as number of participants (%) for categorical variables or as mean ± standard deviation for continuous variables. Between group comparisons performed using Fisher’s exact test for categorical variables or student t-test for continuous variables. For multivariate analysis, results are expressed as adjusted odd ratio and (95% confidence interval) for categorical variables and as mean ± standard error for continuous variables. Comparisons performed using logistic regression for categorical variables and analysis of variance for continuous variables. Multivariate models adjusted on age, body mass index and CRP.

**Supplementary table 4.** Sample size needed to consider the beta coefficient between a bone and blood marker as significant, OsteoLaus study, Lausanne, Switzerland.

|  | | **TBS-BMI** | | **BMD T-score lumbar spine** | | **BMD T-score total hip** | | |
| --- | --- | --- | --- | --- | --- | --- | --- | --- |
|  | | **Coefficient** | **Sample size** | **Coefficient** | **Sample size** | | **Coefficient** | **Sample size** |
| **First assessment** |  | |  |  |  | |  |  |
| Haemoglobin | | -0.007 | 160,179 | -0.022 | 16,215 | | 0.008 | 122,637 |
| Erythrocytes | | -0.021 | 17,796 | -0.075 | - | | -0.031 | 8,166 |
| Leucocytes | | -0.019 | 21,740 | -0.003 | 872,094 | | 0.008 | 122,637 |
| Platelets | | -0.049 | 3,267 | -0.030 | 8,719 | | -0.028 | 10,010 |
| Neutrophils | | 0.048 | 3,405 | 0.035 | 6,405 | | 0.047 | 3,551 |
| Lymphocytes | | -0.120 | - | -0.075 | - | | -0.070 | 1,600 |
| Monocytes | | -0.055 | 2,593 | -0.001 | 7,848,859 | | -0.014 | 40,043 |
| Basophils | | -0.044 | 4,052 | -0.010 | 78,487 | | -0.005 | 313,953 |
| Eosinophils | | -0.002 | 1,962,213 | 0.024 | 13,625 | | 0.038 | 5,434 |
| **Second assessment** | |  |  |  |  | |  |  |
| Haemoglobin | | -0.072 | 1,512 | -0.026 | 11,609 | | -0.037 | 5,732 |
| Erythrocytes | | -0.077 | - | -0.040 | 4,904 | | -0.025 | 12,556 |
| Leucocytes | | -0.139 | - | 0.003 | 872,094 | | -0.027 | 10,765 |
| Platelets | | -0.001 | 7,848,859 | 0.025 | 12,556 | | -0.004 | 490,552 |
| Neutrophils | | -0.088 | - | 0.025 | 12,556 | | -0.015 | 34,882 |
| Lymphocytes | | -0.158 | - | -0.047 | 3,551 | | -0.047 | 3,551 |
| Monocytes | | -0.056 | 2,501 | -0.041 | 4,667 | | -0.016 | 30,658 |
| Basophils | | -0.056 | 2,501 | -0.067 | 1,747 | | -0.095 | - |
| Eosinophils | | -0.029 | 9,331 | 0.060 | 2,179 | | 0.050 | 3,138 |

TBS-BMI, trabecular bone score, adjusted for body mass index; Sample size for significant (p<0.05) coefficients is not provided.

**Supplementary table 5.** Review of the available literature regarding correlation between bone health parameters and blood cell counts in osteoporotic participants.

*(Displays on next page)*

-: not reported, DXA: Dual X-ray absorptiometry, BMD: bone mineral density, pQCT : peripheral quantitative computerized tomography; NS, not significant; HRT: Hormone Replacement Therapy

| **Ref.** | **N** | **Type of study, gender, age selection, country** | **Bone measurements, method** | **Bone health** | **Age  (year)** | **Differences with OsteoLaus** | **Hemoglobin** | **Leucocytes** | **Platelets** | **Neutrophils** | **Lymphocytes** | **Monocytes** | **Eosinophils or Basophiles** |
| --- | --- | --- | --- | --- | --- | --- | --- | --- | --- | --- | --- | --- | --- |
| Kristjansdottir - 2021^(49)^ | 1005 | Prospective, men, Sweden | Bone density  DXA scan | Lumbar spine L1-L4 BMD (g/cm2) 1.12  Total hip BMD (g/cm2) 0.96 | 75.3 | Men included | Positive | NS | Negative | Negative | - | - | - |
| Kim- 2020 ^(48)^ | 8493 | Prospective, men and women, <70yo; Korea | Ultrasound bone densitometry, T-score DXA scan | 21.96% osteoporosis, 49.02% osteopenia | 61.8 | Men included | - | - | Negative | - | - | - | - |
| Valderrabano -2018 ^(35)^ | 1513 | Prospective, men and women, > 65 yo, USA | Bone density  DXA scan | TH T-score average  -1.32 | 74.3 | Men included | NS | Positive | NS | - | - | - | - |
| Schyrr - 2017 ^(50)^ | 143 | Retrospective breast cancer cohort, Switzerland | TBS, T-score  DXA scan | 32% osteoporotic, 45 % osteopenia | 52.1 | Active cancer treatment | NS | - | Positive | Positive | - | - | - |
| Valderrabano - 2016 ^(45)^ | 2589 | Prospective, older men, USA | Bone density  DXA scan | Total hip BMD 0.96 Lumbar spine BMD 1.27 | 78.9 | Men only | Positive | - | Negative | Negative | Positive | Positive and negative | - |
| Lin - 2016 ^(46)^ | 2325 | Retrospective, men, >50 yo , China | Bone density  DXA scan | 24.47% osteopenia or more | 58.5 | Men only | Positive | - | Negative | Negative | NS | Negative | NS |
| Öztürk – 2013 ^(44)^ | 1635 | Retrospective, elderly men and women, Turkey | T-score DXA scan | 53.64 % osteoporosis, 38.17% osteopenia | 72 | Men included | Positive | NS | - | - | - | - | - |
| Korkmaz – 2011 ^(41)^ | 371 | Retrospective, postmenopausal women, Turkey | Bone density  DXA scan | Femur T-score -1.13, Spine T-score -1.43 | 59.7 | Avg BMI > 30 kg/m2,  Diabetes 17.25 %;  Anemia 22% | Positive | - | - | - | - | - | - |
| Kim - 2011 ^(47)^ | 338 | Retrospective, postmenopausal women, Korea | Bone density  DXA scan | T-score  -0.98; 49.4% osteopenia, 5% osteoporosis | 61.2 | Excluded women on HRT  (BMI 22.9±2.8  Diabetes 6.5%) | Positive | Positive | Positive | - | NS | - | - |
| Breuil - 2010 ^(40)^ | 50 | Retrospective, postmenopausal women, France | Bone density  DXA scan | 52% osteoporosis | 71 | Excluded women on HRT | - | NS |  | - | NS for total lymphocytes  Positive for B/CD4 cell subsets | NS | - |
| Laudisio – 2009 ^(42)^ | 358 | Prospective, men and women, > 75 yo, Italy | Ultrasound bone densitometry | T-score  -2.07 | 79.2 | Men included | Positive | - | - | - | - | - | - |
| Cesari - 2005 ^(43)^ | 950 | Prospective, men and women, elderly, Italy | Bone density  right-leg pQCT | At 38% of the tibial length, Total bone density (mg/cm3) 876.81 | 75 | Men included | Positive | - | - | - | - | - | - |
| Di Monaco - 2004 ^(38)^ | 124 | Retrospective, postmenopausal women, Italy | T-score  DXA scan | T-score femoral neck -2.06 | 69.4 | HRT or VitD excluded (BMI 26 +/-4.6SD) | - | - | - | - | Positive | - | - |
| Di Monaco - 2002 ^(39)^ | 176 | Retrospective, postmenopausal women, Italy | T-score  DXA scan | Hip-fractured women, quantitative data not available | 79.8 | All post-fracture  (BMI 23.1+/-4.0SD) | - | - | - | - | Positive | - | - |
